# Usefulness of the ultrathin endoscope with a newly developed knife for complex esophageal endoscopic submucosal dissection

**DOI:** 10.1016/j.vgie.2023.01.006

**Published:** 2023-03-12

**Authors:** Satoki Shichijo, Muneaki Miyake, Ryu Ishihara

**Affiliations:** 1 Department of Gastrointestinal Oncology, Osaka International Cancer Institute, Osaka, Japan

## Abstract

Video 1 Usefulness of an ultrathin endoscope with a newly developed knife for complex esophageal endoscopic submucosal dissection

Usefulness of an ultrathin endoscope with a newly developed knife for complex esophageal endoscopic submucosal dissection

A 62-year-old woman underwent subtotal esophagectomy for esophageal cancer, local resection of pharyngeal cancer with postoperative radiotherapy, and bilateral cervical lymph node dissection owing to pharyngeal cancer metastasis. A follow-up endoscopy revealed a 10-mm brownish area in the cervical esophagus, consisting of type B1 vessels according to the magnifying endoscopic classification of the Japan Esophageal Society,^1^ and the biopsy revealed squamous intraepithelial neoplasia.

Endoscopic submucosal dissection (ESD) was performed with the patient under general anesthesia because the lesion was located in the cervical esophagus (Video 1, available online at www.giejournal.org). We started marking with a magnifying endoscope (GIF-H290Z; Olympus, Tokyo, Japan); however, we could not mark the depressed area (Fig. 1). Thus, we switched to a colonoscope (PCF-H290TI; Olympus), which had a wide down angle and completed circumferential markings (Figs. 2 and 3). Next, we performed a mucosal incision followed by submucosal dissection using a clip and line (Fig. 4) to obtain a good visibility of the submucosal layer and to facilitate efficient submucosal dissection. However, we could not dissect the posterior and left sides because of the restricted maneuverability of the scope due to previous surgery and radiotherapy. We then switched to an ultrathin endoscope (GIF-XP290N; Olympus) with a newly developed knife (Endosaber Fine; Sumitomo Bakelite Co, Ltd, Tokyo, Japan) and completed dissection (Fig. 5) with increased maneuverability of the scope, resulting in en bloc resection of the specimen (Fig. 6) without any adverse events. The final pathological diagnosis was squamous cell carcinoma (Fig. 7) without lymphovascular invasion, with negative vertical margins and indeterminate horizontal margins. Follow-up endoscopy 1 year after ESD revealed no local recurrence without stricture (Fig. 8).

**Figure 1:**
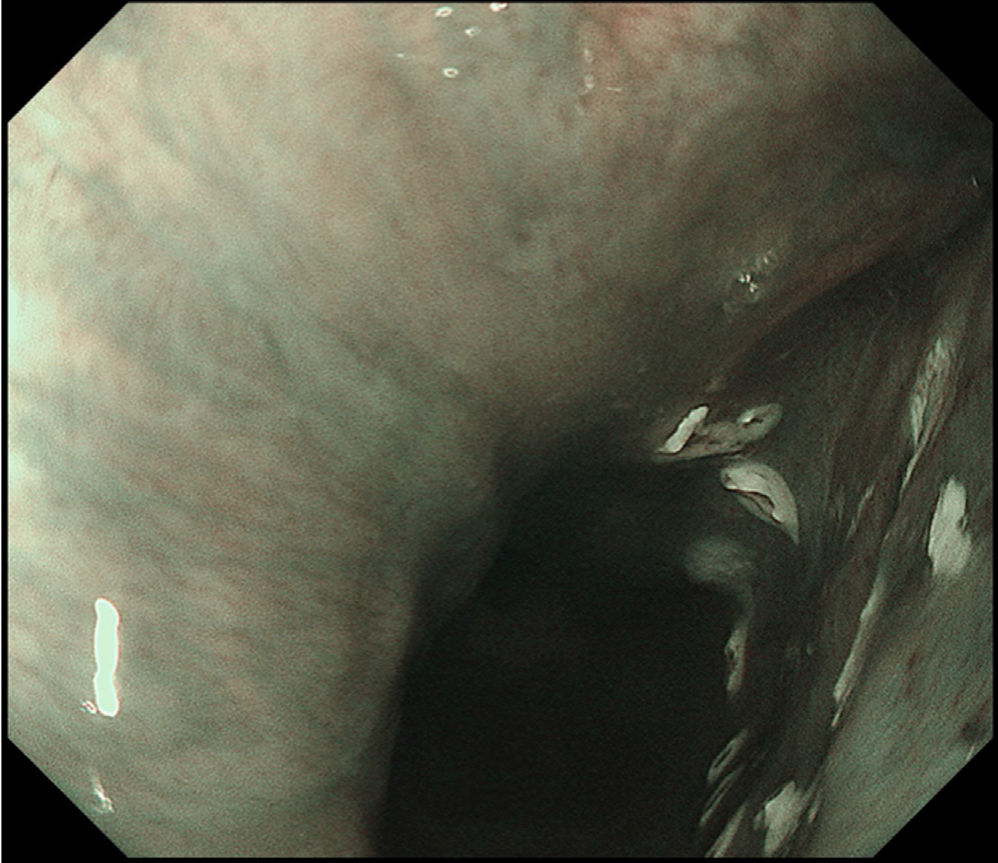
The lesion was partially located at the depressed part of the remnant esophagus.

**Figure 2:**
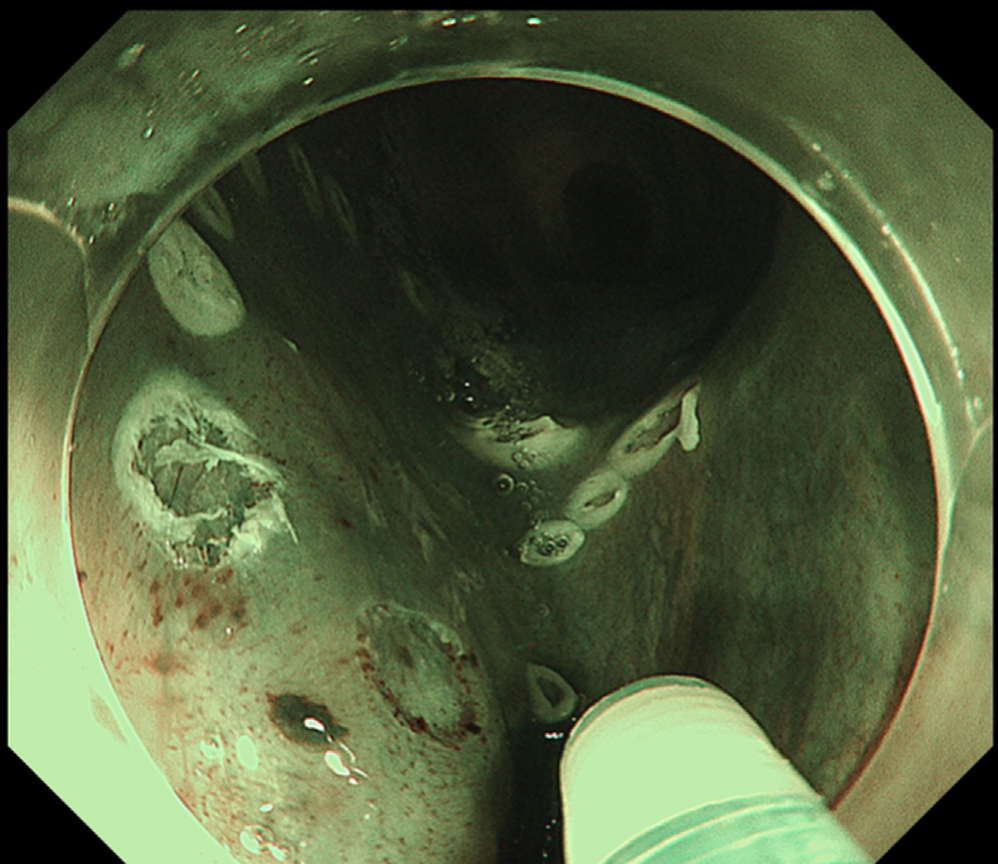
Marking with a colonoscope at the depressed area.

**Figure 3:**
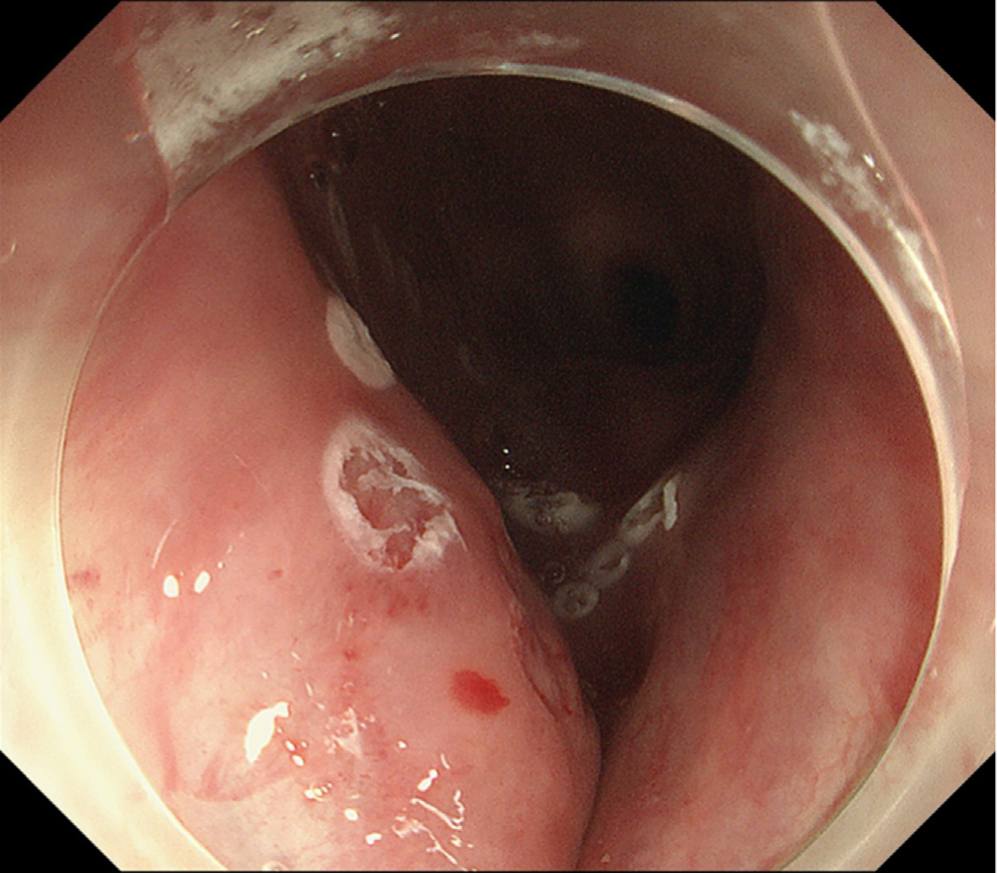
Circumferential markings were completed.

**Figure 4:**
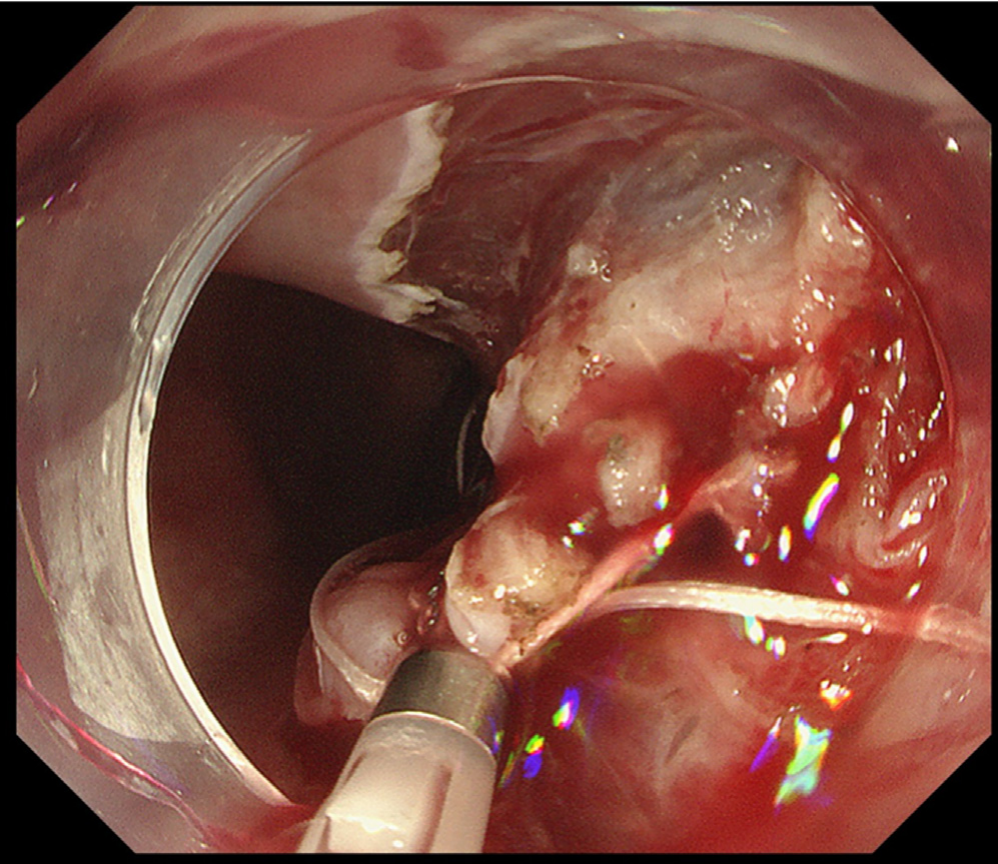
Clip with the line was attached to the specimen.

**Figure 5:**
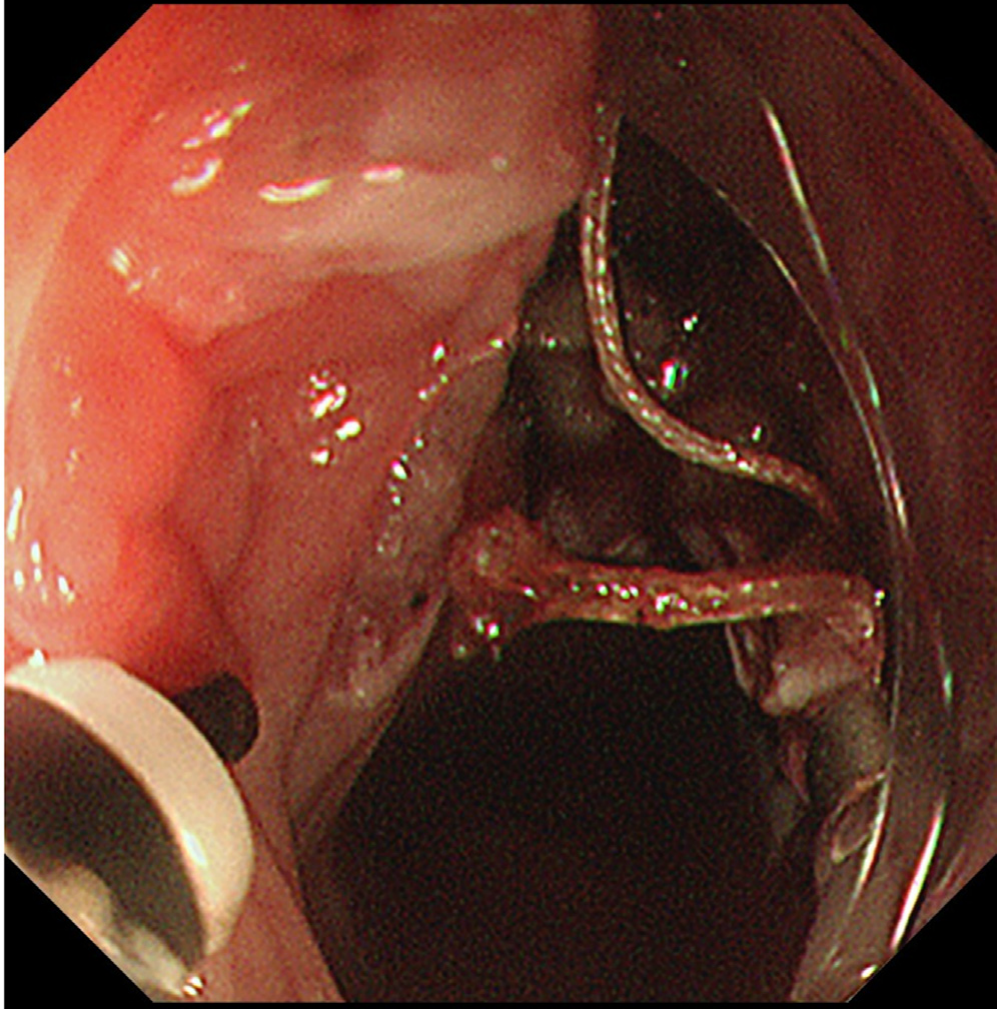
Endoscopic submucosal dissection with an ultrathin endoscope and a thin knife.

**Figure 6:**
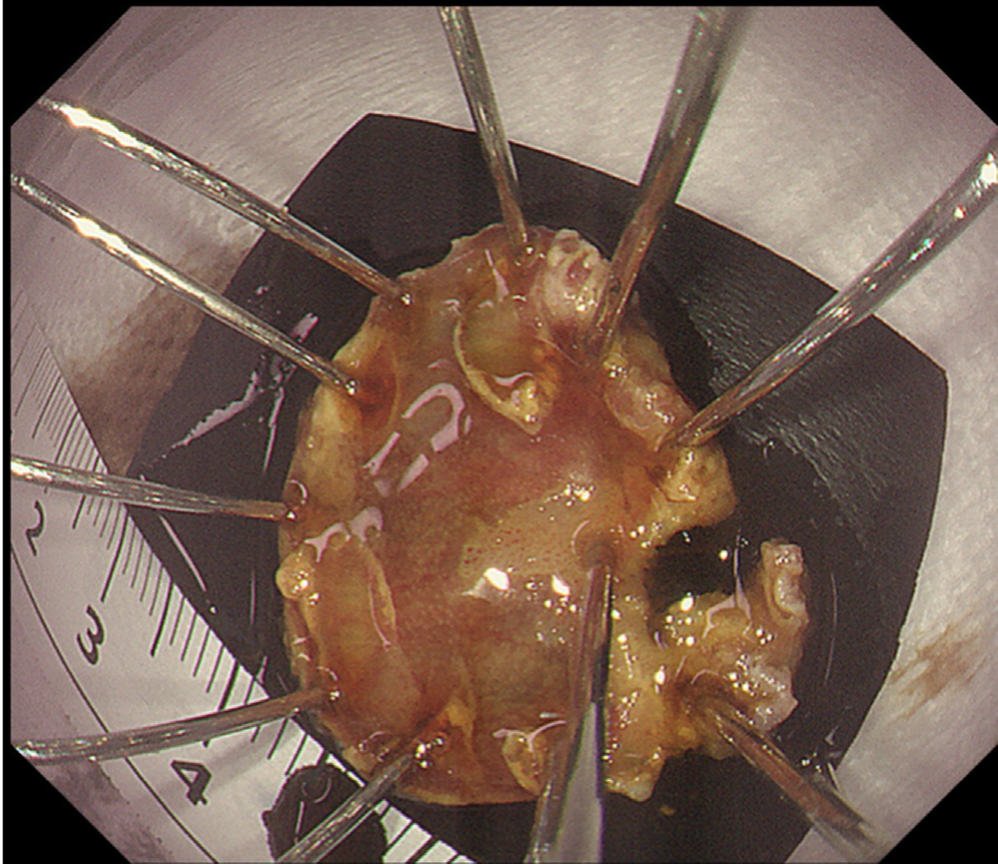
The specimen was resected en bloc.

**Figure 7:**
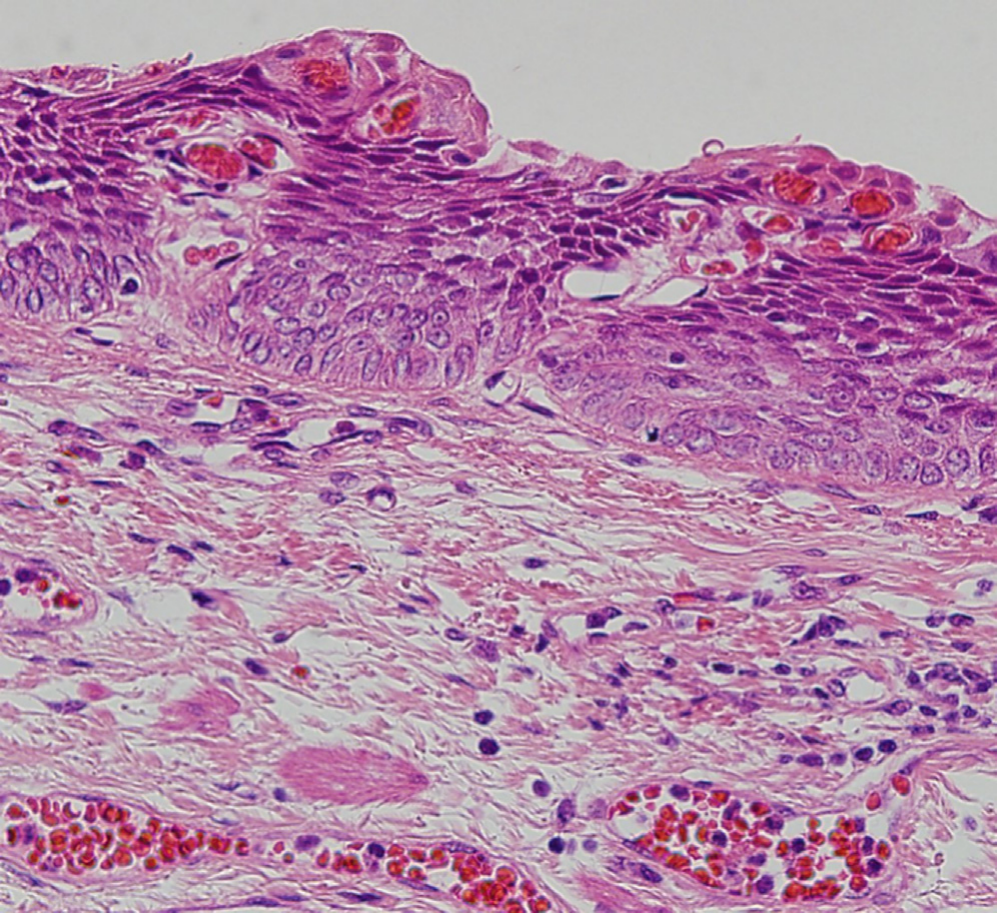
The final pathological diagnosis was squamous cell carcinoma without lymphovascular invasion (H&E, orig. mag. ×400).

**Figure 8:**
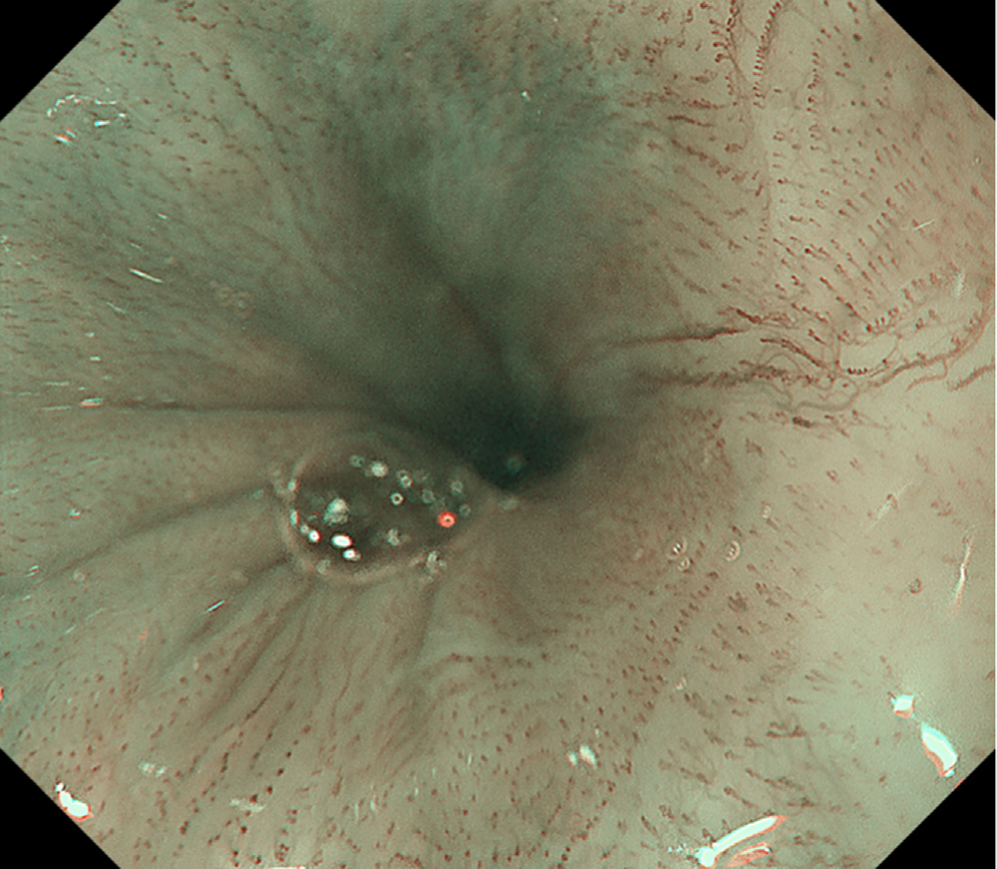
The follow-up endoscopy 1 year after endoscopic submucosal dissection revealed no local recurrence.

The usefulness of ESD with an ultrathin endoscope with the tip of a snare for esophageal strictures has been reported.^2^^,^^3^ The ultrathin endoscope GIF-XP290N can operate in a narrow space as its channel diameter is as small as 2.2 mm. The newly developed Endosaber Fine (Fig. 9), with its thin sheets of 1.95-mm width, can be accepted in the small working channel of 2.2 mm and used together with an ultrathin endoscope.^4^^,^^5^ We performed an ESD at a complex location with the ultrathin endoscope and the newly developed knife together with a handmade transparent “hood,” which was made of a transparent sheath.

**Figure 9:**
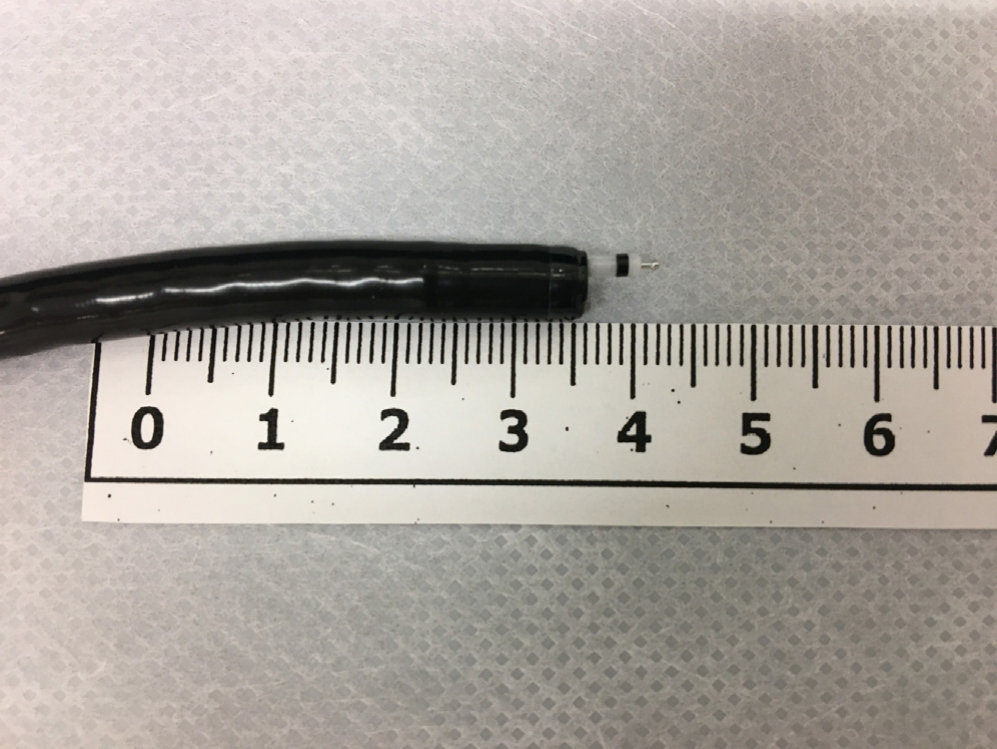
The newly developed Endosaber Fine, a needle-type knife whose tip is fixed to the sheath, inserted through the ultrathin endoscope.

## Supplementary data

Video 1 Usefulness of an ultrathin endoscope with a newly developed knife for complex esophageal endoscopic submucosal dissection
